# Comparative Analysis of Amorphous and Biodegradable Copolymers: A Molecular Dynamics Study Using a Multi-Technique Approach

**DOI:** 10.3390/molecules30051175

**Published:** 2025-03-06

**Authors:** Alovidin Nazirov, Jacek Klinowski, John Nobleman

**Affiliations:** 1 Department of Macromolecular Physics, Adam Mickiewicz University, ul. Umultowska 85, 61-614 Poznań, Poland; 2 Department of Chemistry, University of Cambridge, Lensfield Road, Cambridge CB2 1EW, UK; jk18@cam.ac.uk; 3 Natural Science Department, LaGuardia Community College, City University of New York, 31-10 Thomson Ave, Long Island City, NY 11101, USA

**Keywords:** glycolide, lactide, caprolactone, biopolymer, molecular dynamics, relaxation spectroscopy, ^1^H and ^13^C solid-state NMR, DSC, FTI

## Abstract

We investigate the molecular dynamics of glycolide/lactide/caprolactone (Gly/Lac/Cap) copolymers using differential scanning calorimetry (DSC), Fourier transform infrared spectroscopy (FTIR), ^1^H second-moment, ^1^H spin-lattice relaxation time (T_1_) analysis, and ^13^C solid-state NMR over a temperature range of 100–413 K. Activation energies and correlation times of the biopolymer chains were determined. At low temperatures, relaxation is governed by the anisotropic threefold reorientation of methyl (-CH_3_) groups in lactide. A notable change in T_1_ at ~270 K and 294 K suggests a transition in amorphous phase mobility due to translational diffusion, while a second relaxation minimum (222–312 K) is linked to CH_2_ group dynamics influenced by caprolactone. The activation energy increases from 5.9 kJ/mol (methyl motion) to 22–33 kJ/mol (segmental motion) as the caprolactone content rises, enhancing the molecular mobility. Conversely, lactide restricts motion by limiting rotational freedom, thereby slowing global dynamics. DSC confirms that increasing ε-caprolactone lowers the glass transition temperature, whereas higher glycolide and lactide content raises it. The onset temperature of main-chain molecular motion varies with the composition, with greater ε-caprolactone content enhancing flexibility. These findings highlight the role of composition in tuning relaxation behavior and molecular mobility in copolymers.

## 1. Introduction

Biodegradable polymers made from glycolide (Gly), lactide (Lac), and caprolactone (Cap) are widely used in medicine for applications such as drug carriers, tissue engineering scaffolds, and temporary surgical implants. These copolymers [1,2,3], synthesized with metal catalyst initiators, offer enhanced properties compared to pure polyesters such as polyhydroxybutyrate (PHB), polylactide (PL), polyglycolide (PGL), and polycaprolactone (PCL) [4,5]. The key advantage of poly(Gly-Lac-Cap) is its tunable molecular dynamics, which vary significantly based on the monomer concentrations in the polymer chain. The sequence and ratio of monomers directly influence the polymer’s physical and chemical properties, such as the mechanical strength, degradation rate, and biocompatibility. Molecular dynamics—such as the crystallinity, chain flexibility, and interactions between the monomers—play a crucial role in determining how the copolymer behaves in a biological environment. By controlling these dynamics through synthesis conditions, the copolymer’s performance can be optimized for specific medical applications, offering tailored solutions for controlled drug release, tissue engineering, and bioresorbable medical devices. In our previous molecular dynamics investigation [6] of a binary monomer chain system composed of Gly and Lac, we utilized differential scanning calorimetry (DSC) and proton (^1^H) nuclear magnetic resonance (NMR) to confirm that the synthesized copolymer systems are homogeneous and amorphous. The results indicated that an amorphous miscible phase was formed, without introducing significant structural heterogeneities or defects. Nozirov et al. [7] demonstrated that molecular motion in copolymer sequences of Gly and Lac (e.g., 20Gly/80Lac, 50Gly/50Lac, and 80Gly/20Lac) is influenced by the degrees of freedom of the Gly and Lac units.

In addition, our previous [7] study showed that in copolymer sequences of glycolide (Gly) and caprolactone (Cap), increasing the Gly content in a caprolactone copolymer significantly influences the designed properties. As the Gly content increases, it disrupts chain molecular packing and introduces defects, leading to greater chain flexibility. These defects result in a decrease in the copolymer’s melting temperature compared to pure polycaprolactone (PCL), as Gly introduces more amorphous regions and reduces the order of crystallinity. Additionally, the glass transition temperature (T_g_) typically lowers, making the copolymer more flexible at lower temperatures. Conversely, increasing the Cap content makes the polymer chains more rigid because the longer Cap units promote more ordered molecular interactions and stronger chain packing, ultimately elevating the melting temperature. The flexibility observed in the copolymer is also associated with segmental conformational changes, primarily in the glycolide units, which require an activation energy of 44 kJ/mol. Lower-temperature methyl group rotations contribute to relaxation motions with an activation energy of approximately 11 kJ/mol. Thus, the balance between Gly and Cap content in the copolymer governs its overall behavior. In copolymers with a high Gly content, such as 0.8Gly/0.2Cap, the chain flexibility increases, leading to lower thermal stability. However, as the Gly content decreases and the Cap content increases (e.g., 0.2Gly/0.8Cap), the polymer becomes more rigid, with a higher melting temperature and reduced flexibility. This balance between amorphous flexibility and crystalline rigidity, along with the accompanying changes in activation energy for molecular motions, provides a versatile approach to tailoring the properties of the copolymer.

The purpose of this study is to gain a deeper understanding of the molecular motion mechanisms responsible for the physical and chemical properties of these materials. The dynamics of active molecular groups, investigated through various solid-state NMR techniques, provide insights into relaxation mechanisms such as translational molecular motion and segmental molecular displacements. Solid-state NMR techniques, including the ^1^H second-moment resonance line, ^1^H spin-lattice relaxation times, and ^13^C MAS NMR, allow the investigation of a broad range of fluctuation mechanisms [8] and molecular motions in deliberately designed synthetic copolymers [9].

The copolymers studied consist of Gly, Lac, and Cap units randomly linked in the polymer chains. Chain modulation, involving adjustments in the monomer ratio or arrangement, is key to designing biodegradable materials for medical applications. By altering monomer composition, we can control physical properties like the mechanical strength, degradation rate, crystallinity, and biodegradability. Molecular dynamics investigations provide insights into how these structural changes affect material behavior, enabling the design of tailored materials for drug delivery, tissue engineering, and surgical implants.

The key objective is to provide chemical engineers with valuable information that can help optimize the copolymers’ thermal stability, mechanical properties, and degradation rates, enabling tailored solutions for specific applications and enhancing their value for advanced manufacturing and sustainable alternatives to traditional plastics.

## 2. Results

**Figure 1:**
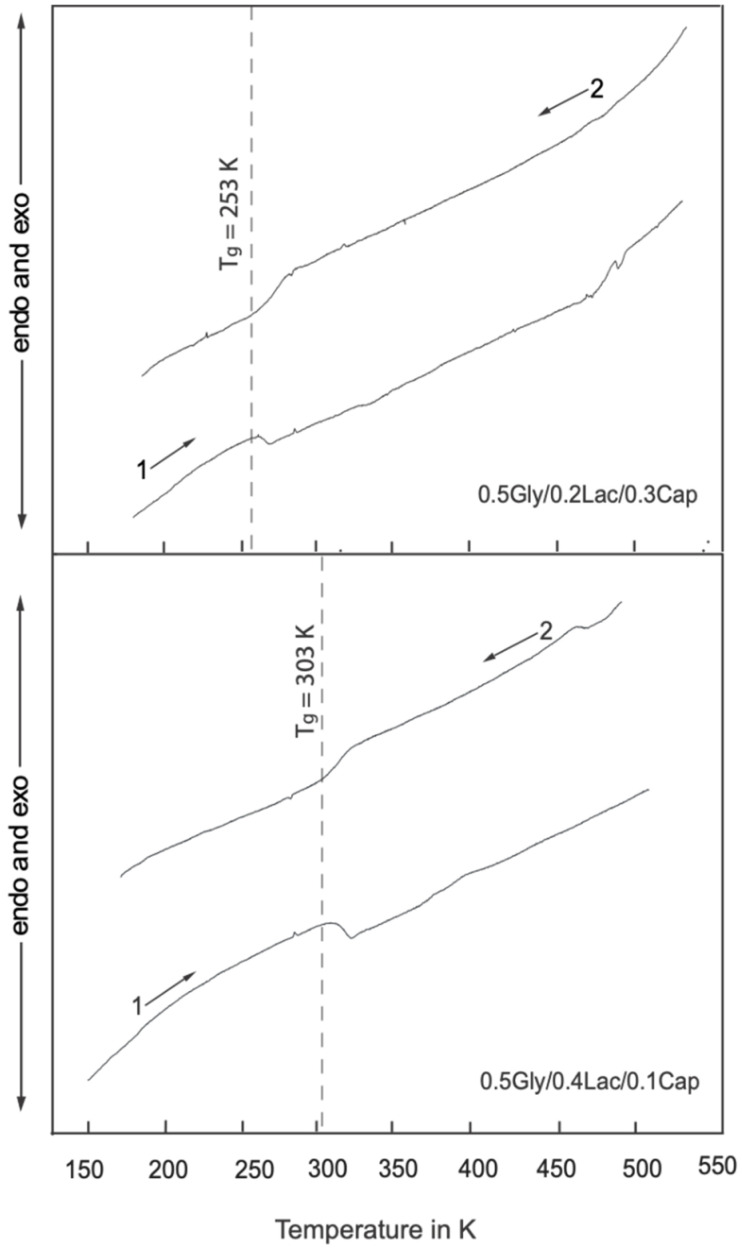
DSC thermograms of copolymers 0.5Gly/0.2Lac/0.3Cap and 0.5Gly/0.4Lac/0.1Cap, where heating (1⟶) and cooling (⟵2) are indicated. The dashed vertical lines represent a phase transition temperature (T_g_).

Figure 1 shows the DSC thermograms of both Gly/Lac/Cap copolymers in the temperature range of 160 K to 550 K. The vertical lines indicate the glass transition temperatures (Tg), which are 253 K for 0.5Gly/0.2Lac/0.3Cap and 303 K for 0.5Gly/0.4Lac/0.1Cap. The absence of broad endothermic and exothermic crystallization peaks during the heating (1→) and cooling (←2) processes suggests that both synthesized copolymers are homogeneous. The dashed vertical lines (---) represent the averaged glass transition temperature (T_g_). The shift in the glass transition temperature to higher values with increasing lactide content strongly indicates its influence on thermal behavior, while a higher concentration of caprolactone lowers the glass transition temperature (see Figure 1). The DSC thermograms confirm that the synthesized polymers are amorphous, with no detectable crystalline phases in either copolymer.

**Figure 2:**
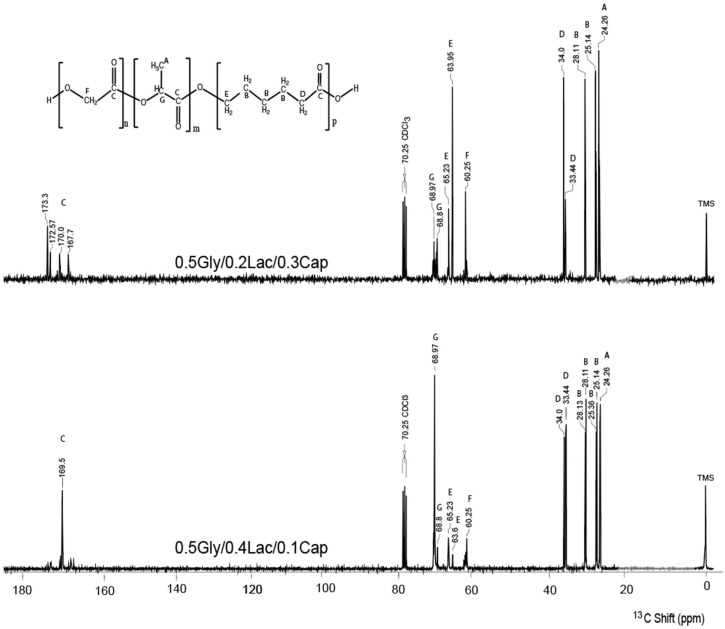
Solution-state ^13^C NMR spectra (75 MHz) of copolymers 0.5Gly/0.2Lac/0.3Cap and 0.5Gly/0.4Lac/0.1Cap recorded at 313 K. Letters (A–G) indicate the assignment of molecular groups. The samples were dissolved in CDCl_3_ (chloroform-d, 77.2 ppm, triplet due to deuterium coupling), with TMS (0 ppm) as the internal reference.

Figure 2 shows the high-resolution solution-state ^13^C NMR spectra of 0.5Gly/0.2Lac/0.3Cap and 0.5Gly/0.4Lac/0.1Cap at 313 K, with the chemical structure sequence residues illustrated and molecular groups assigned using letters (A–E). Both spectra exhibit strong resonance signals for the methyl (-CH_3_) groups of the lactide unit in the range of 7 to 17.34 ppm, followed by sharp signals corresponding to methylene (-CH_2_-) and methine (-CH-) groups between 40 and 60 ppm. Additionally, high-frequency signals in the range of 150 to 157 ppm are attributed to the carbonyl (C=O) groups of the Gly-Lac-Cap copolymer.

**Figure 3:**
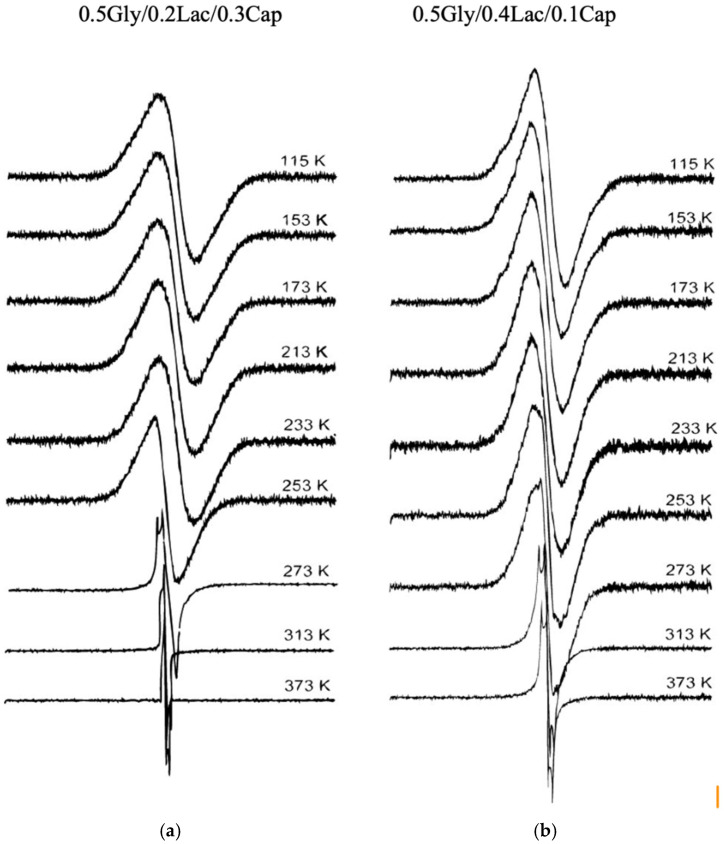
Derivatives of ^1^H NMR absorption spectra of (**a**) 0.5Gly/0.2Lac/0.3Cap and (**b**) 0.5Gly/0.4Lac/0.1Cap at different temperatures. The proton derivative spectra at 273 K are highly sensitive compared to the broad DSC lines (c.f. Figure 1) for both polymers, indicating the early onset of chain molecular dynamics motion in preparation for the phase transition.

Figure 3a,b shows derivatives of the ^1^H NMR absorption spectra of 0.5Gly/0.2Lac/0.3Cap and 0.5Gly/0.4Lac/0.1Cap at different temperatures. The proton derivative spectra at 273 K are highly sensitive compared to the broad DSC lines (c.f. Figure 1) for both polymers, indicating the early onset of chain molecular dynamics motion in preparation for the phase transition. At low temperature (115 K), both copolymers exhibit a stiff amorphous structure, with relaxation occurring through methyl molecular motion. At 250 K, additional derivatives indicate the onset of trans-gauche segmental motion, marking the transition from a quasi-stable state towards a more mobile phase, which is further confirmed by the changes observed in DSC, proton second moment analysis, and the mono-exponential growth of proton T_1_ relaxation.

**Figure 4:**
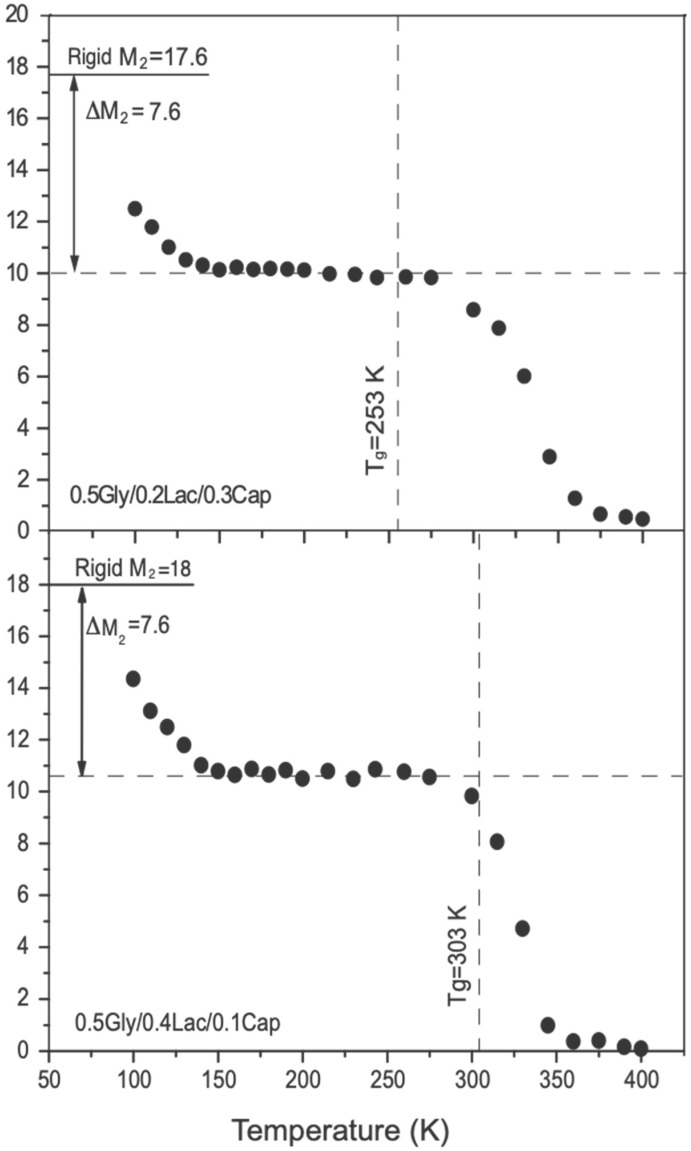
Second moments of the ^1^H NMR lines of 0.5Gly/0.2Lac/0.3Cap and 0.5Gly/0.4Lac/0.1Cap versus temperature. The glass phase transition temperatures, T_g_ indicated by the vertical lines (according to DSC, c.f. Figure 1).

Figure 4 shows the second moment (*M*_2_) of ^1^H for 0.5Gly/0.2Lac/0.3Cap and 0.5Gly/0.4Lac/0.1Cap as a function of temperature. At low temperatures (e.g., 90 K), the measured *M*_2_ values are 0.129 and 0.138 mT^2^. As the temperature increases to 130 K, *M*_2_ decreases to 0.102, and 0.11 mT^2^ and remains nearly constant in the 130–280 K range. Methyl group reorientation plays a major role at temperatures below 275 K. With a further rise in temperature, *M*_2_ gradually decreases, eventually reaching a plateau of 0.005 mT^2^ at 365 K and 375 K, respectively. Although the second moment *M*_2_ of ^1^H in both samples follows a similar trend, notable differences are observed in the reduction curves at the plateau regions. Commonly, by analyzing the second moment, we can assess the molecular rigidity and differentiate between crystalline and amorphous phases in any polymers. Notably, both our samples exhibit amorphous characteristics, as indicated by DSC, derivatives of ^1^H NMR absorption spectra (c.f. Figure 3), second moment analysis, and the mono-exponential growth of proton T_1_ relaxation. Additionally, the solid-state ^13^C NMR experiments confirmed the reliability of the gathered data, further supporting their amorphous nature.

**Figure 5:**
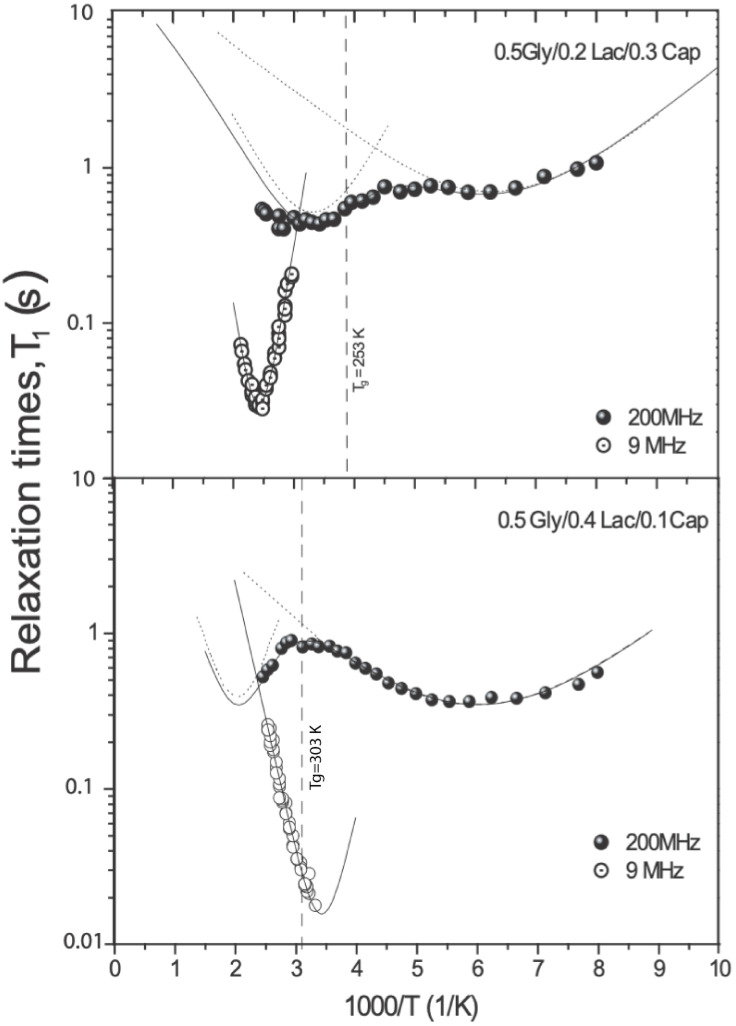
Arrhenius plots of ^1^H spin-lattice relaxation times measurements at 200 MHz and 9 MHz for 0.5Gly/0.2Lac/0.3Cap and 0.5Gly/0.4Lac/0.1Cap. ^1^H experimental data fitted using the BPP model as indicated by solid lines. The experiments conducted from the low to high temperatures and the glass phase transition T_g_ indicated by the vertical dashed lines (according to DSC, c.f. Figure 1).

Figure 5 presents the ^1^H spin-lattice relaxation time (T_1_) measurements for 0.5Gly/0.2Lac/0.3Cap and 0.5Gly/0.4Lac/0.1Cap as a function of temperature, plotted against β = 10^−3^/T K^−1^. The Arrhenius plots were derived from exponential fitting of proton relaxation measurements at 200 MHz and 9 MHz. The T_1_ curves reveal three distinct relaxation modes, corresponding to the proton spin system’s relaxation via methyl (-CH_3_), methylene (-CH_2_-), and complex trans–gauche isomerization motions within the amorphous phases of both copolymers. The figure also illustrates the recovery of ^1^H magnetization for both samples at various temperatures. An exponential recovery was observed for all measurements, consistent with the results of the DSC (c.f. Figure 1), implying the presence of a single type of proton spin-lattice relaxation time (T_1_) in both copolymers. Generally, a single-exponential pattern of spin-lattice relaxation is expected due to sufficient spin diffusion in strongly dipolar-coupled proton systems. However, in cases where the proton coupling system consists of two distinct spatially well-separated regions (e.g., amorphous versus crystalline), the spins in these regions cannot communicate effectively through proton spin diffusion due to differences in motional dynamics. In such cases, biexponential spin-lattice relaxation is observed [10]. All our experimental data were fitted using single-exponential components.

**Figure 6:**
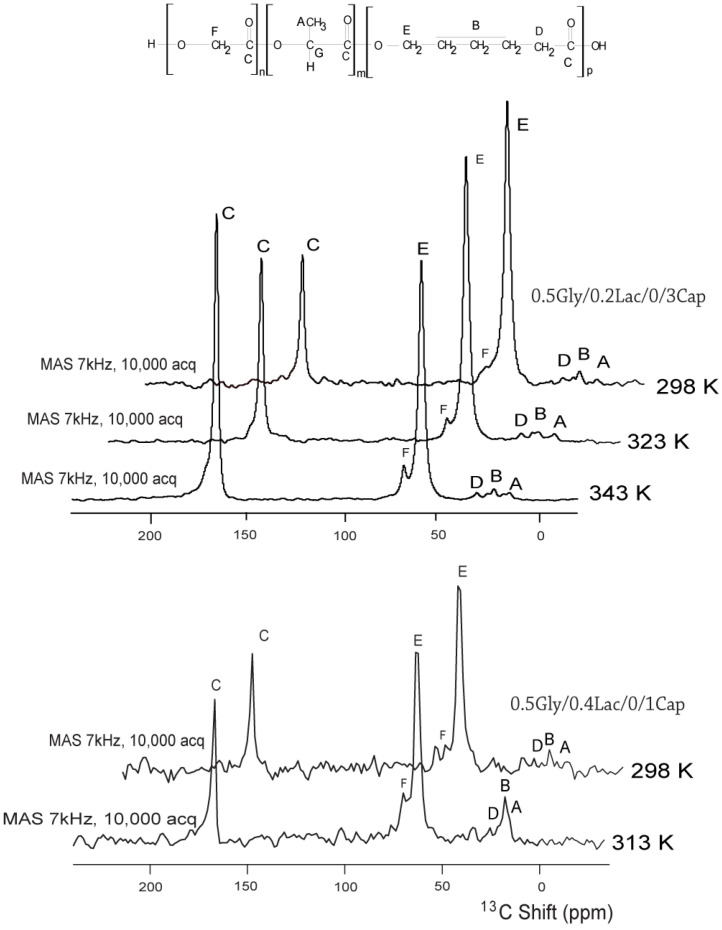
The resonance frequency of 75.56 MHz ^13^C solid-state NMR of copolymers 0.5Gly/0.2Lac/0.3Cap and 0.5Gly/0.4Lac/0.1Cap at different temperatures. The molecular groups are indicated by letters corresponding to the structure of copolymer chains. Solid-state NMR technology is still under development compared to its solution-state counterpart. One of the key technical challenges is the difficulty of spinning amorphous materials at high speeds. Due to their unique behavioral properties, such as superfluid-like elasticity, these materials tend to lose centrifugal axis stability during rotation. The magnitude of ^13^C hydrocarbon signals effectively increases with rising temperature, corresponding to an increase in intensity due to fast trans-gauche isomerization and translational diffusion motion.

Figure 6 presents the ^13^C solid-state NMR spectra of 0.5Gly/0.2Lac/0.3Cap and 0.5Gly/0.4Lac/0.1Cap at different temperatures. At 323 K, the rotor containing the 0.5Gly/0.2Lac/0.3Cap copolymer spun easily. However, the rotor with the 0.5Gly/0.4Lac/0.1Cap sample encountered stability issues during spinning just above 313 K. Multiple attempts were made to improve the stability by reducing the sample amount inside the rotor, but all efforts were unsuccessful. Consequently, the spectrum was excluded from further analysis due to a deteriorated signal-to-noise (S/N) ratio. To understand the cause of these technical difficulties at 323 K, we examined the molecular motion in the Lac-concentrated chain sequences. At this temperature (β = 3.09 K^−1^), the molecular motion reached the endpoint of the phase transition region, where intramolecular interactions were not yet established. As a result, the amorphous polymer behaved like a superfluid with low viscosity. This low-viscosity state prevented the solid-state rotor from spinning properly. Spinning amorphous material in solid-state NMR presents a significant technical challenge. To address this issue, we optimized the experimental conditions to ensure proper spin relaxation, allowing for accurate differentiation of chemical shifts in ppm. To enhance the spectral readability and confirm the peak assignments, we conducted complementary solution-state ^13^C NMR measurements (c.f. Figure 2) to aid in the interpretation of Figure 6 peaks. Overcoming these limitations could pave the way for new possibilities in characterizing fluid-like spin systems using solid-state NMR methods.

**Figure 7:**
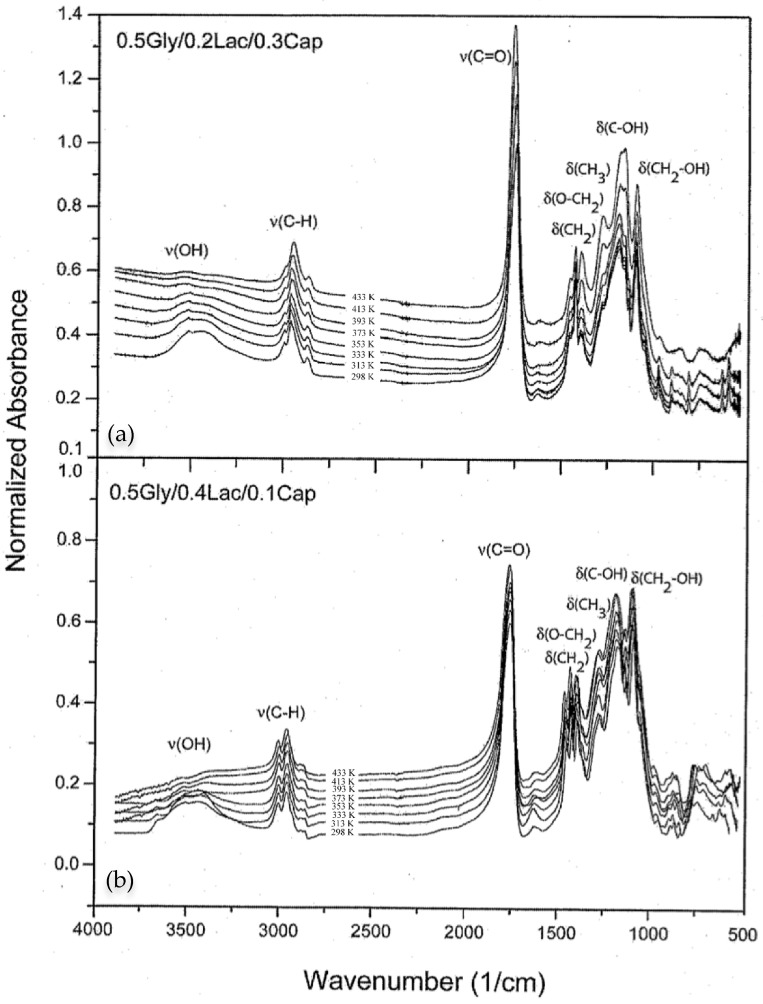
FTIR spectra of copolymers (**a**) 0.5Gly/0.2Lac/0.3Cap and (**b**) 0.5Gly/0.4Lac/0.1Cap at different temperatures. The band assignments correspond to hydroxyl end-groups (-OH), carbonyl (C=O), methyl (-CH_3_), methylene (-CH_2_-), and methide (-CH-) vibrational motions. The spectra clearly indicate differences in the vibrational motions of the modulated chains.

Figure 7 presents the FTIR spectra of 0.5Gly/0.2Lac/0.3Cap and 0.5Gly/0.4Lac/0.1Cap at different temperatures. The spectra show dipole moment vibrations of the hydroxyl (-OH) bond group at 3500 cm^−1^, the C-H (sp^3^) bond between 2750 cm^−1^ and 3000 cm^−1^, and the carbonyl (C=O) ester group within the range of 1720–1750 cm^−1^. As the temperature increases, the vibration mode at 3500 cm^−1^ flattens due to the equal distribution of hydroxyl end-groups motion across the polymer chain segments. The -CH group’s vibration motion, influenced by induced dipole–dipole interactions between hydrocarbons, exhibits minimal peak shifts. Additionally, the symmetric (C=O) vibration mode becomes more pronounced at higher temperatures. While changes in the hydroxyl vibrational motion are observed, the hydroxyl reorientations below (T_g_) contribute insignificantly to proton relaxation mechanisms.

## 3. Discussion

### 3.1. Second Moment M_2_ of ^1^H NMR Lines

The second moment was determined to assess the molecular rigidity and dipole–dipole interactions, helping to distinguish the phases. Notably, for the copolymers 0.5Gly/0.2Lac/0.3Cap (M_w_ = 64,000 g/mol) and 0.5Gly/0.4Lac/0.1Cap (M_w_ = 79,000 g/mol), DSC data (c.f. Figure 1) show that the monomers were evenly incorporated into the chain matrix, forming an amorphous phase. To facilitate the understanding of the molecular dynamics occurring in the Gly/Lac/Cap copolymers, the values of the ^1^H second moment—induced by the motion states through bond distances of the atomic monomers—are presented. For simplicity in the calculations, the rigid lattice value was divided into intra- and inter-molecular contributions, as described in previous studies [11,12]. The intramolecular contribution *M*_2_*^intra^*, according to Van Vleck [13], arises from interactions between nuclei within a molecule and is dependent on the molecular structure, as given by the equation:

(1) $$M_{2}^{intra}=358.1N_{0}^{-1}\underset{i}{\sum }\underset{i\neq 1}{\sum }r^{-6},$$

where *N*_0_ is the number of protons in a molecule, *n_i_* is the number of structurally equivalent protons of type *i* per molecule, and *r* is the distance between the *i*-th and *j*-th protons in Ångströms. The intramolecular contributions were calculated using the positions of protons by minimizing the potential energy of the molecule with the Gaussian program [14]. After minimizing the potential energy of the chain, which includes specifically modulated monomers in the structure, the minimized polymer chain structure was input into a custom-made program based on the Van Vleck theory which was used to calculate the second moment (*M*_2_). Following this, the molecular contributions were calculated based on the positions of these protons, and the results are presented in Table 1.

In our calculations, two different motion states in the molecule were considered: the reorientation of the methyl groups around their three-fold symmetry (C_3_) and the reorientation of the hydroxyl groups around their two-fold symmetry (C_2_), which we previously studied in polyhydroxybutyrate and polylactide. For the rigid molecules of 0.5Gly/0.2Lac/0.3Cap and 0.5Gly/0.4Lac/0.1Cap, the *M*_2_ values were calculated as 0.176 mT^2^ and 0.18 mT^2^, respectively. When the methyl reorientation was considered, the calculated *M*_2_ values decreased to 0.107 mT^2^ and 0.11 mT^2^. Furthermore, when the hydroxyl reorientation was taken into account, as observed in pure polylactide, the values of *M*_2_ obtained were 0.097 mT^2^. Comparing this to 0.102 mT^2^, the hydroxyl group contribution is very small (about 0.01 mT^2^), which is imperceptible through the second-moment change. However, the increase in lactide monomer concentration from 0.2% to 0.4% contributed to the position of the plateau at a level of 0.11 mT^2^. The (-CH_2_-) groups of glycol and (-CH-) lactide protons made a relatively small contribution to *M*_2_ due to the rigidity of their structure, and such small contributions are neglected in further discussion. Our previous study shows that in copolymers such as 0.2Gly/0.8Cap and 0.8Gly/0.2Cap, the reduction in the *M*_2_ value at low temperatures is due to methylene local oscillations and small trans-gauche isomerization motions in their chains, which lead to an insignificantly small reduction (ca. 0.01 mT^2^). At high temperatures, these samples showed a reduction in the *M*_2_ value due to free global molecular tumbling motion and the self-diffusion process.

Interestingly, in both samples of 0.5Gly/0.2Lac/0.3Cap and 0.5Gly/0.4Lac/0.1Cap, the small quantity of lactide monomers hindered tumbling motions and significantly slowed the free global motions. In the temperature range from 250 K to 370 K, the *M*_2_ value of 0.5Gly/0.2Lac/0.3Cap decreased gradually from 0.10 to 0.05 mT^2^, and the *M*_2_ value of 0.5Gly/0.4Lac/0.1Cap decreased from 0.11 to 0.05 mT^2^.

This regular reduction in *M*_2_ behavior indicates the involvement of a complex hindered dynamic, ultimately converging into the general molecular reorientation motion. Table 1 lists the experimental and calculated second-moment values for polyglycolide, polylactide, polycaprolactone, and the studied copolymers for comparison. The average second-moment reduction, resulting from all molecular motions present in the copolymers, was calculated to be 16.26 mT^2^ for 0.5Gly/0.2Lac/0.3Cap and 16.33 mT^2^ for 0.5Gly/0.4Lac/0.1Cap. These values indicate strong competition between the lactide and caprolactone concentrations in constraining the dynamic motion of the chains. When comparing these values with those of polyglycolide (21 mT^2^), polylactide (15.8 mT^2^), and polycaprolactone (23 mT^2^), it is evident that the lactide monomers form hydrogen bonds with methyl groups. These bonded chains hinder rotational motions, while caprolactone, which has more -CH_2_- groups, increases the induced dipole moment along the chains. The high dipole moment in the chain tends to overcome the barrier of the potential energy associated with hindered motion during trans-gauche isomerization rotational motions. Broadband dielectric relaxation studies on pure caprolactone showed that at low temperatures, local mobility corresponds to the *γ*β relaxation, while at high temperatures, the *γ*β modes reflect the segmental mobility of the chains [15].

The intermolecular contribution to the proton second moment, due to interactions between nuclei in neighboring molecules, is given by [16]

(2) $$M_{2}^{intra}=358.1(4\pi N_{o}\rho 3R^{3}1.66M),$$

where *ρ* is the density in g/cm^3^, *R* is the molecular radius in Å, and *M* refers to the molecular weight in atomic units (a.u.). For the polylactide sample studied here, *ρ* = 1.276 g/cm^3^, *R* = 3 Å, and *M* = 149 a.u. Therefore, the calculated *M*_2_ value is 0.031 mT^2^. We used the Smith method [17,18] to calculate the intermolecular contributions for easier comparison, as listed in Table 1. The intermolecular calculations for polycaprolactone and polyglycolide were neglected in further studies due to their insignificant contribution to the second-moment values. Figure 4 shows the measured *M*_2_ values: approximately 0.130 mT^2^ and 0.138 mT^2^ at 90 K and 0.10 ± 0.01 mT^2^ in the temperature range of 130 K to 275 K. By comparing the experimental data with the calculated results, we can reasonably conclude that methyl group reorientation plays a major role at temperatures below 275 K. Theoretical calculations indicate that the contribution of hydroxyl reorientations to the *M*_2_ value is approximately 0.01 mT^2^, which falls within the error bars of the measured *M*_2_ values. Thus, the measured *M*_2_ values are not sensitive to hydroxyl reorientation occurring in the temperature range of 275 K to 350 K.

### 3.2. ^1^H Spin-Lattice Relaxation Times

^1^H spin-lattice relaxation in Gly/Lac/Cap is entirely governed by proton dipolar interactions between randomly sequenced monomers of molecular groups, modulated by reorientational motions, and consequently involving a single correlation time. The proton spin-lattice relaxation rate (1/*T*_1_^*H*^) is described by the Bloembergen–Purcell–Pound (BPP) model [19,20]. The BPP theory describes nuclear spin relaxation in terms of molecular motion, linking the spin-lattice relaxation time (*T*_1_) to the spectral density function (e.g., correlation time over frequency) of molecular fluctuations, according to the following formula:

(3) $$\frac{1}{T_{1}^{H}}=C\left(\frac{\tau_{C}}{1+\tau_{C}^{2}\omega_{0}^{2}}+\frac{4\tau_{C}}{1+4\tau_{C}^{2}\omega_{0}^{2}}\right),$$

where *τ_C_* is the correlation time describing the dynamic process, *ω*₀ is the Larmor angular frequency, and *C* is a dipole–dipole relaxation constant. This constant is associated with proton distances and is also proportional to the reduction of the second moment (∆*M*_2_) induced by molecular motions, as given by

(4) $$C=\frac{2}{3}\Delta M_{2}\gamma_{H}^{2},$$

where *γ^H^* is the proton gyromagnetic ratio.

For thermally activated diffusion, the correlation time *τ_C_* is typically described by the Arrhenius equation:

(5) $$\tau_{c}=\tau_{o}exp\frac{E_{a}}{RT}$$

where *τ_o_* is the pre-exponential factor corresponding to the rotational correlation time at infinite temperature, *R* is the gas constant, *T* is the absolute temperature (K), and *E_a_* is the activation energy (per mole) for the molecular dynamic process. Table 2 lists all parameters obtained from the fittings in Figure 5.

Figure 5 shows the relaxation behavior of 0.5Gly/0.2Lac/0.3Cap and 0.5Gly/0.4Lac/0.1Cap. Both methyl groups of the copolymers exhibit the first motion, as described by Equations (3) and (5), with activation energy and ***C*** parameters having similar values, as indicated by the solid and dashed lines. Well-defined minima are observed in the temperature range of 130 K (β = 7.69 K^−1^) to 270 K (β = 3.7 K^−1^), with spin-lattice relaxation times of 0.69 s and 0.45 s. For these minima, the *M*_2_ values derived from Equation (4) were 0.10 and 0.109 mT^2^, showing good agreement with the intra-methyl reduction motion.

The second distribution minima, occurring between 222 K (β = 4.5 K^−1^) and 312 K (β = 3.2 K^−1^), are associated with the methylene (-CH_2_-) groups, where the addition of caprolactone significantly influences the relaxation mechanism. At higher temperatures, complex dynamic motions lead to the relaxation of copolymer chains. The main segmental motion of the chains begins at 312.5 K (β = 3.2 K^−1^), though it is difficult to observe at high fields (200 MHz). However, in the 9 MHz experiment, this motion corresponds to a minimum of approximately 29 ms at 400 K (β = 2.5 K^−1^) for 0.5Gly/0.2Lac/0.3Cap and about 20 ms at 294 K (β = 3.4 K^−1^) for 0.5Gly/0.4Lac/0.1Cap.

The discontinuities in T_1_ at approximately 270 K (β = 3.7 K^−1^) and 294 K (β = 3.4 K^−1^) are attributed to changes in molecular dynamics within the amorphous phase, particularly due to the onset of translational diffusion. These findings are consistent with ^13^C MAS NMR experimental observations (c.f. Figure 6). The ^13^C resonances in 0.5Gly/0.2Lac/0.3Cap at room temperature are notably narrower than those in 0.5Gly/0.4Lac/0.1Cap, implying that the latter compound has lower flexibility. The magnitude of ^13^C hydrocarbon signals effectively increases with rising temperature, corresponding to an increase in intensity due to fast trans-gauche isomerization and translational diffusion motion. These motions are strongly reflected in the ^1^H relaxation measurements at 9 MHz.

### 3.3. Identification of the Functional Groups by FTIR

Figure 7 illustrates the Fourier transform infrared spectroscopy (FTIR) spectra of 0.5Gly/0.2Lac/0.3Cap and 0.5Gly/0.4Lac/0.1Cap at different temperatures. The dipole moment vibrations of the hydroxyl (-OH) bond at 3500 cm^−1^, the -CH bond at 2750–3000 cm^−1^, and the carbonyl (C=O) bond at 1750 cm^−1^ are observed. As the temperature increases, the copolymers’ vibration modes at 3500 cm^−1^ flatten due to the equal distribution of terminal hydroxyl groups, with rotational motion occurring in the segments of the chain. The deformation vibrations of O-CH_2_ groups at 1470–1435 cm^−1^ become more pronounced with increasing temperature. In the frequency range of 1350–1000 cm^−1^, the stretching vibration of the C-OH bond occurs, while at 1125–992 cm^−1^, medium absorption bands are observed due to the deformation vibrations of CH_2_-OH groups. The dipole vibration of the -CH group, due to its strong dipole–dipole interaction with hydrocarbons, hardly shifts its absorbance peak. The C=O dipole vibration is symmetric; thus, at high temperatures, a sharp mode is observed. Changes in the vibration motion of the hydroxyl group are further examined in molecular dynamics simulations. The Gaussian program allows the assignment of frequency types for the oscillation motions, as presented in Table 3, which lists the experimental and calculated frequency parameters for the specific molecular groups occurring in these copolymers.

As the temperature increases, the infrared vibration frequency band at 1470–1435 cm^−1^, corresponding to the O-CH_2_ groups with a maximum at 1421 cm^−1^, shifts toward a higher wavenumber. Similarly, the band at 1100–1060 cm^−1^, corresponding to the oscillations of the CH_2_-OH group, with a maximum at 1090 cm^−1^, moves to a lower vibrational frequency. Calculations show the maximum frequencies at 1484 and 1090 cm^−1^ for both vibrational modes. The shift in position primarily corresponds to fluctuations in the molecular forces during vibration. If the force is constant, and the energy of the CH_2_-OH molecular bond decreases with increasing temperature, this indicates free group vibrational motion. The magnitude and position of the absorption wavenumber for the O-CH_2_ groups strongly depend on the molar concentration of the caprolactone monomer. An increase in temperature allows for more freedom in vibration. If the molar concentration of caprolactone exceeds 0.3%, the wavenumber shifts toward a lower frequency, allowing the O-CH_2_ group to oscillate more freely. If the molar concentration of caprolactone is less than 0.1%, the force of vibration remains constant and the energy of interaction increases, which hinders free oscillation and leads to bond rigidity.

## 4. Experiments

### 4.1. Sample Synthesis

The monomers of glycolide (Boehringer Ingelheim, Ingelheim, Germany) and L-lactide (Aldrich Corp., Steinheim, Germany) were used as supplied without further purification. *ϵ*-caprolactone (Fluka, Buchs, Switzerland) was dried and distilled under argon before the synthesis reaction [21]. Copolymerization of Gly/Lac/Cap in the molar ratios 0.5Gly/0.2Lac/0.3Cap and 0.5Gly/0.4Lac/0.1Cap was carried out in bulk at 373 K using zirconium (IV) acetylacetonate as the initiator [20]. The products were degassed and sealed under vacuum. They were then ground, shaken with methyl alcohol to remove unreacted monomers, and dried under vacuum at 323 K. Liquid chromatography (LC) using a Waters ALC/GPC 3m apparatus provided the average molecular weights: 0.5Gly/0.2Lac/0.3Cap, M_w_ = 64,000 g/mol, and 0.5Gly/0.4Lac/0.1Cap, M_w_ = 79,000 g/mol. Tetrahydrofuran (THF) was used as the aprotic solvent for elution, and polystyrene standards were employed for calibration.

### 4.2. Differential Scanning Calorimetry (DSC)

The molecular weights of the copolymer samples were determined, with each sample (5 mg) packed into sealed aluminum pans and placed in a Netzsch-204 DSC. The heating and cooling temperatures were scanned at a rate of 10 K/min. Initially, the samples were cooled to 160 K and held isothermally for 5 min. The temperature was then gradually increased to 550 K, held isothermally for 1 min, and subsequently scanned back to the initial temperature of 160 K. The chamber was purged with argon gas, and liquid nitrogen was used as the cooling agent. This cycle was repeated twice, and the glass transition temperature (T_g_) of each sample was accurately determined.

### 4.3. NMR Spectroscopy

The solution ^13^C NMR spectra were recorded on a Varian Unity Inova spectrometer with a superconducting magnet providing a 7.05 T field strength and a 75 MHz carbon frequency. A small amount of the sample was dissolved in CDCl_3_, with tetramethylsilane (TMS) used as an internal standard. The spectra were obtained at 313 K with 32 scans, a 3.74 s acquisition time, and a 7 ms pulse width. Chloroform-d (CDCl_3_) was used as the solvent, with TMS calibrated at 0 ppm.

#### 4.3.1. ^1^H NMR Second Moment Measurement

Samples for ^1^H solid-state NMR were sealed in glass ampoules after being degassed under vacuum at 0.01 Pa for 48 h at room temperature to remove paramagnetic oxygen and traces of water, thereby eliminating the effects of paramagnetic impurities on longitudinal relaxation times. Derivatives of the NMR absorption spectra were recorded at 25 MHz using a continuous-wave NMR spectrometer of the marginal oscillator type. Figure 3 and Figure 4 show the derivatives of the ^1^H NMR absorption spectra measured at different temperatures. Second moments (*M*_2_) were calculated from the spectra (c.f Figure 3) by numerical integration. The dependence of *M*_2_ on temperature is shown in Figure 3 and Figure 4 for 0.5Gly/0.2Lac/0.3Cap and 0.5Gly/0.4Lac/0.1Cap, respectively.

#### 4.3.2. ^1^H NMR Spin-Lattice Relaxation Time (T_1_) Measurement at 200 MHz

^1^H spin-lattice relaxation times (T_1_) were measured at a Larmor frequency of 200 MHz using a Bruker CXP NMR spectrometer with a saturation recovery sequence. The measurements were performed in the aliphatic region, specifically at 1.3 ppm, corresponding to the methylene (−CH_2_−) protons. The acquired free induction decays (FIDs) were always monoexponential and used to estimate the T_1_ values presented in Figure 5, with an uncertainty of no more than 5%. The sample temperature was controlled using a gas-flow cryostat and monitored with an accuracy of 1 K.

#### 4.3.3. ^1^H NMR Spin-Lattice Relaxation Time (T_1_) Measurement at 9 MHz

^1^H T1 relaxation times at a Larmor frequency of 9 MHz were measured using a commercial fast field cycling (FFC) spectrometer, Stelar Spinmaster 2000. For measurements at 9 MHz, the cycling technique with a pre-polarizing field was employed. Magnetization recovery was always found to be exponential, and errors in T1 measurements were estimated to be approximately 5%. The sample temperature was controlled via a gas-flow cryostat and monitored with a calibrated Pt resistance thermometer, ensuring an accuracy of 1 K. The 9 MHz measurement corresponds to low-field proton NMR, which is particularly sensitive to molecular motion in the intermediate motional regime, providing insights into slow dynamics and relaxation processes. The 200 MHz measurement, conducted using high-field NMR, allows detailed chemical shift analysis and helps differentiate between various local environments within the polymer structure. The combination of these two frequencies enables a comprehensive study of molecular mobility and relaxation mechanisms.

#### 4.3.4. Solid-State ^13^C NMR Experiments

Solid-state ^13^C NMR experiments were performed on a Bruker Advance NMR spectrometer operating at 75.56 MHz, using a high-power decoupling sequence with proton decoupling at 75 kHz. The π/2 pulse length was 3 µs. The amorphous copolymer sample was packed in a 4 mm zirconium rotor, sealed with an O-ring cap, and spun at the magic angle (7 kHz). The acquisition time was 0.01 s, the repetition time was 5 s, and 10,000 scans were acquired to achieve a good signal-to-noise (S/N) ratio. The sample temperature was controlled by a gas-flow cryostat and monitored using a calibrated Pt resistance thermometer with an accuracy of 0.5 K. The spectra of both copolymers were acquired at room temperature.

### 4.4. Fourier Transform Infrared (FTIR) Spectroscopy

FTIR absorption spectra of the copolymers 0.5Gly/0.2Lac/0.3Cap and 0.5Gly/0.4Lac/0.1Cap were measured as a function of temperature, ranging from 298 K to 498 K with a 20 K step, using a Bruker IFS66/S spectrometer. The temperature was controlled automatically by a heat cell with a platinum thermocouple, providing an accuracy of ± 0.1 K. Spectra were recorded in the frequency range of 4000–500 cm^−1^ with a resolution of 4 cm^−1^ and 16 scans, using the standard KBr pellet technique. The pellets consisted of 200 mg of dry KBr powder and 1 mg of copolymer, pressed at a pressure of 4 tons/cm^2^.

## 5. Conclusions

The experiments and calculations show that, at low temperatures, the primary mechanism driving relaxation is the anisotropic reorientation of methyl (-CH_3_) groups in lactide. This insight highlights the distinct molecular dynamics and relaxation behaviors between pure homopolymers and alternating copolymers. The differential scanning calorimetry (DSC) results confirm that the glass transition temperature (T_g_) decreases as the molar ratio of ε-caprolactone increases, suggesting a more homogeneous amorphous structure with higher chain mobility. In contrast, an increase in glycolide and lactide content raises T_g_, likely due to weak molecular interactions involving the oxygen carbonyl groups, which reduce chain mobility.

Second-moment values for the rigid lattice reveal that, at low temperatures, relaxation occurs through reorientation of the (-CH_3_) groups around their threefold symmetry axes, reducing dipolar contributions. The temperature dependence of the spin-lattice relaxation time (T_1_) is strongly influenced by copolymer composition, with distinct T_1_ minima corresponding to -CH_3_ group rotations when the molar fraction of L-lactide is ≥0.2. For copolymers with ≥0.3 ε-caprolactone content, additional minima emerge, reflecting main-chain molecular motions. The low-temperature minima are attributed to CH_3_ group reorientation, while the high-temperature minima correspond to trans-gauche segmental motions of the polymer chain. The onset temperature of main-chain motion, which is composition-dependent, indicates that a higher ε-caprolactone content enhances the polymer elasticity.

## Figures and Tables

**Table 1:** Calculated and experimental values of the ^1^H second moments of copolymers of 0.5Gly/0.2Lac/0.3Cap and 0.5Gly/0.4Lac/0.1Cap.

Motion	*M*_2_ Calculated (×10^2^ mT^2^)	*M*_2_ Experimental (×10^2^ mT^2^)
Stationary molecule of polyglycolide	11	11 at 90 K
Stationary molecule of polylactide	15.8	
Stationary molecule of polycaprolactone	23.5	
Stationary molecule of 0.5Gly/0.2Lac/0.3Cap	17.6	12.8 at 90 K
CH3 (intra-inter C_3_), reduction of PLLA	10.7 − 3.1 = 7.6	10.9 intra at 130 K
Reduction of the chains motion	9.5	9.5 at 340 K
Average	16.26	10.83
Stationary molecule of 0.5Gly/0.4Lac/0.1Cap	18	13.8 at 90 K
CH/intra-inter > (C_3_), reduction of PLLA	10.7 − 3.1 = 7.6	10.9 intra at 130 K
Reduction of the chains motion	9.5	9.9 at 340 K
Average	16.33	11.22

**Table 2:** Motional parameters obtained from the ^1^H relaxation measurements.

^1^H Relaxation Parameters			
**0.5Gly/0.2 Lac/0.3Cap**	**I—Motion at 200 MHz**	**II—Motion at 200 MHz**	**III—Motion at 9 MHz**
c (×10^9^s^−2^)	2.6	3.5	5.26
*τ*_o_ (s)	6.0 × 10^−12^	1.7 × 10^−12^	1.3 × 10^−14^
E (kJ/mol)	5.9	14.2	33
**0.5Gly/0.4Lac/0.1Cap**	**I—Motion at 200 MHz**	-	**III—Motion at 9 MHz**
c (×10^9^s^−2^)	5.3	-	3.58
*τ*_o_ (s)	9.5 × 10^−11^	-	1.0 × 10^−13^
E (kJ/mol)	5.9	-	22

**Table 3:** The calculated IR ranges corresponding to the vibrations of molecular groups in the copolymers of Gly/Lac/Cap.

IR Range (cm^−1^)	Type of Bond	Intensity
3650–3200	*ν*(-OH)	Weak
3000–2800	*ν*(sp^3^(C-H))	Weak
1720–1680	*ν*(C=O)	Strong
1480–1450	δ(CH_2_)	Medium
1470–1435	δ(O-CH_2_)	Medium
1394–1040	δ(CH_3_)	Medium
1350–1000	δ(C-OH)	Medium
1100–1060	δ(CH_2_-OH)	Medium

## Data Availability

The original contributions presented in this study are included in the article. Further inquiries can be directed to the corresponding author(s).

## References

[1] Kricheldolf H.R., Rost S. (2005). A-B-A-Triblock and multiblock copolyesters prepared from ε-caprolactone, glycolide and l-lactide by means of bismuth subsalicylate. Polymer.

[2] Goldberg E.P., Nakajima A. (1980). Biomedical Polymers: Polymeric Materials and Pharmaceuticals for Biomedical Use.

[3] Chiellini E., Giusti P. (1983). Polymers in Medicine: Biomedical and Pharmacological Applications.

[4] Nobleman J. (2021). A Study of Polymer Dynamics by Solid-State NMR.

[5] Kariduraganavar M.Y., Kittur A.A., Kamble R.R., Kumbar S.G., Laurencin C.T., Deng M. (2014). Polymer Synthesis and Processing. Natural and Synthetic Biomedical Polymers.

[6] Nozirov F., Fojud Z., Jancelewicz M., Nazirov A., Jurga S. (2008). Molecular Motion in the Biocopolymer Sequence of Glycolide and Lactide Studied by Solid-State NMR. Appl. Magn. Reson..

[7] Nozirov F., Szczesniak E., Fojud Z., Dobrzynski P., Klinowski J., Jurga S. (2002). ¹H and ¹³C NMR Studies of Molecular Dynamics in the Biocopolymer of Glycolide and ε-Caprolactone. Solid State Nucl. Magn. Reson..

[8] Nobleman J. (2020). Solid State NMR Spectroscopy Study of Molecular Dynamics of Pyridine 1-Oxide Encapsulated in p-tert-Butylcalix[4]arene. J. Phys. Chem. A.

[9] Dobies M., Makrocka-Rydzyk M., Jenczyk J., Jarek M., Spontak R.J., Jurga S. (2017). Molecular Dynamics Study of Polystyrene-b-Poly(ethylene oxide) Asymmetric Diblock Copolymer Systems. Langmuir.

[10] Lee K.W., Lee C.E., Choi J.Y., Kim J. (2005). Distinct Critical Fluctuations and Molecular Motions Manifest in a Model Biomembrane. Solid State Commun..

[11] McBrierty V.J., Packer K.J. (2006). Nuclear Magnetic Resonance in Solid Polymers.

[12] Andrew E.R. (1956). Nuclear Magnetic Resonance.

[13] Van Vleck J.H. (1948). The Dipolar Broadening of Magnetic Resonance Lines in Crystals. Phys. Rev..

[14] Frisch M.J., Trucks G.W., Schlegel H.B., Scuseria G.E., Robb M.A., Cheeseman J.R., Zakrzewski V.G., Montgomery J.A., Stratmann R.E., Burant J.C. (1998). Gaussian 98, Revision A.7.

[15] Grimau M., Laredo E., Pérez Y. M.C., Bello A. (2001). Study of dielectric relaxation modes in poly(ε-caprolactone): Molecular weight, water sorption, and merging effects. J. Chem. Phys..

[16] Abragam A. (1983). The Principles of Nuclear Magnetism.

[17] Smith G.W. (1965). Proton Magnetic Resonance Studies of Solid Triethylenediamine—Molecular Structure and Motions. J. Chem. Phys..

[18] Smith G.W. (1965). Proton Magnetic Resonance Studies of Solid Tetramethyls of Silicon, Germanium, Tin, and Lead. J. Chem. Phys..

[19] Bloembergen N., Purcell E.M., Pound R.V. (1948). Relaxation Effects in Nuclear Magnetic Resonance Absorption. Phys. Rev..

[20] Beckmann P.A. (1988). Spectral densities and nuclear spin relaxation in solids. Phys. Rep..

[21] Dobrzynski P., Suming L., Kasperczyk J., Janeczek H., Bero M., Gasc F., Vert M. (2005). Structure-Property Relationships of Copolymers Obtained by Ring-Opening Polymerization of Glycolide and E-Caprolactone. Part 1. Synthesis and Characterization. Biomacromolecules.

